# Ultrasonographic Assessment of Anatomic Relationship Between the Internal Jugular Vein and the Common Carotid Artery in Infants and Children After ETT or LMA Insertion: A Prospective Observational Study

**DOI:** 10.3389/fped.2020.605762

**Published:** 2020-10-29

**Authors:** Yipeng Du, Jin Wang, Limin Jin, Chunping Li, Haichun Ma, Su Dong

**Affiliations:** ^1^Department of Anesthesiology, The First Hospital of Jilin University, Changchun, China; ^2^Department of Urology, The First Hospital of Jilin University, Changchun, China

**Keywords:** Internal Jugular Vein (IJV), common carotid artery, laryngeal mask airway, infants and children, ultrasound

## Abstract

**Background:** Central venous catheterization is used for fluid management and infusion of drugs, but it is difficult to perform and carries a high incidence of complications in infants and children. In adults, the anatomic relationship and the overlap index between the internal jugular vein (IJV) and the common carotid artery (CCA) changed significantly after laryngeal mask airway (LMA) placement. However, there are conflicting results regarding the anatomic relationship between the IJV and the CCA after endotracheal tube (ETT) or LMA insertion in pediatric populations.

**Aim:** The aim of this study was to compare the overlap index and anatomic relationship between the IJV and the CCA in infants and children after ETT or LMA insertion by ultrasonography.

**Method:** This single-center, prospective, observational study including 92 infants and children, aged 1 month to 6 years, were grouped according to the airway devices placed: Group ETT (*n* = 44) and Group LMA (*n* = 48). The overlap index and anatomic relationship between the IJV and the CCA before and after airway device insertion at neutral and 30° head rotation position were evaluated by ultrasonography.

**Results:** Before airway device insertion, as the head was rotated 30° to the contralateral side, the overlap index increased significantly on the right side of the neck compared to the neutral head position. In Group ETT, there was no significant difference in the overlap index after intubation in the neutral head position or 30° head rotated position on either side. In Group LMA, the overlap indexes were increased significantly after LMA insertion in the neutral head position on both sides. Likewise, the overlap indexes were increased significantly after LMA insertion in the 30° head rotated position on both sides. The most common positional relationship between the IJV and the CCA was anterolateral (AL) in both the right side and left side in the neutral head position. In Group ETT, the AL position was still the most common position relationship between the IJV and the CCA before and after intubation in the 30° head rotated position. In Group LMA, the anterior (A) position increased significantly after LMA insertion in the left side. In the 30° head rotated position, there was a significant increase to the A position after LMA insertion in both the right side and left side. The change from AL to A was increased after LMA insertion, especially in the 30° head rotated position.

**Conclusions:** The overlap indexes of the IJV and the CCA increased significantly in both sides of the neck after LMA placement in the neutral head position, especially in 30° head rotated position. The IJVs after LMA placement had a tendency to become anterior to the CCA when the head of the patient rotated to the opposite direction in infants and children.

## Introduction

Catheterization of the central venous is an important procedure in pediatric patients undergoing major surgery because it allows for fluid management, central venous pressure measurement, administration of vasoactive drug therapy, and blood sampling (1). The internal jugular vein (IJV) is frequently used for central venous catheterization because its lower incidence of serious complications such as arterial puncture, hemothorax, pneumothorax or airways compromises (2). Technically, IJV catheterization is generally considered more difficult to perform and carries a high incidence of complications in infants and children due to anatomical variations, small size of the vein, and tendency of the vessel to collapse (3, 4).

Although the use of ultrasound assistance can increase the success rate and reduce the incidence of complications has been shown in pediatric patients (5), ultrasound devices are not always available. Without the ultrasound assistant, IJV catheterization is generally performed using anatomical landmark technique or pulsation of the common carotid artery (CCA). Anatomical variation and overlapping of the CCA by the IJV increased the probability of accidental arterial puncture, which is the most common complication related to IJV catheterization (6).

In clinical practice, endotracheal tube (ETT) and laryngeal mask airway (LMA) are two common airway devices for airway management during general anesthesia. It has been reported that the anatomic relationship between the IJV and the CCA changed significantly and the degree of overlap between the IJV and the CCA also increased in adults after LMA placement (7). However, there are conflicting results regarding the anatomic relationship between the IJV and the CCA after ETT or LMA insertion in pediatric populations (8–10). Therefore, we believe that it is important for us to investigate this topic.

The purpose of this study was to compare the anatomic relationship and degree of overlap between the IJV and the CCA in infants and children after ETT or LMA insertion by ultrasonography.

## Methods

After obtaining approval from the ethics committee of The First Hospital of Jilin University and written informed parental consent, 101 infants and children, aged 1 month to 6 years of ASA physical status I or II and scheduled for elective surgery (urological surgery, general surgery, and plastic surgery) under general anesthesia, were included in this study. Exclusion criteria included previous trauma or surgery involving the neck, previous IJV catheterization, and known or suspected abnormal neck anatomy.

The patients were grouped according to the airway devices used: Group ETT or Group LMA. The patients were standard monitored. After inhalational or intravenous induction of anesthesia, patients were hand ventilated using a facemask. Patients were placed supine in a 15° Trendelenburg position with the head in a neutral position. The head and the neck were slightly extended by placing a roll under the center of the upper back. The size of the roll was adjusted to the patients and not to be larger than the size of the neck.

Ultrasound measurements of the neck were performed using a Mindray DC-8 Elite (Mindray Medical, Shenzhen, China) with a 9-MHz linear array probe. The probe was placed perpendicular to the skin, and images of the left and right IJV and CCA were obtained at the cricoid cartilage level. Minimal pressure was applied to reduce the compression of the IJV as best as we can. After freezing the image on the ultrasound screen, we selected the image showing the largest cross-sectional area (CSA) at the end of inspiration to eliminate the respiratory effect then rotated the head of the patient 30° to the contralateral side and obtained the images again.

Following this, the ETT or LMA was placed with the head in a neutral position. The cuffs of both ETT and LMA were well-inflated without air leak. The sizes of ETT for patients 1–3 months, 3–9 months, and 9–24 months were 3.0–3.5, 3.5–4.0, and 4.0–4.5, respectively. The size of ETT for patients more than 24 months was selected using the formula [age (in years)/4 + 4]. The sizes of LMA were selected according to the weight of patients (1: <5 kg, 1.5: 5–10 kg, 2: 10–20 kg, 2.5: 20–30 kg). Mechanical ventilation was set with tidal volume 10 mL/kg, and the respiratory rate was adjusted to maintain the end-expiratory partial pressure of carbon dioxide between 35 and 45 mmHg, without application of positive end-expiratory pressure. Anesthesia was maintained using propofol and sevoflurane 1.0–2.0% in an oxygen/air mixture. After establishing artificial airways, patients were re-positioned as before (supine in a 15° Trendelenburg position with the head in a neutral position and rotated 30° to the contralateral side) and ultrasound images were re-recorded.

The same investigator manipulated the ultrasound probe and stored the images in the ultrasound machine in order to standardize measurements. All data were measured on the ultrasound screen by another investigator later who was blinded to the inserted airway device.

**Figure 1:**
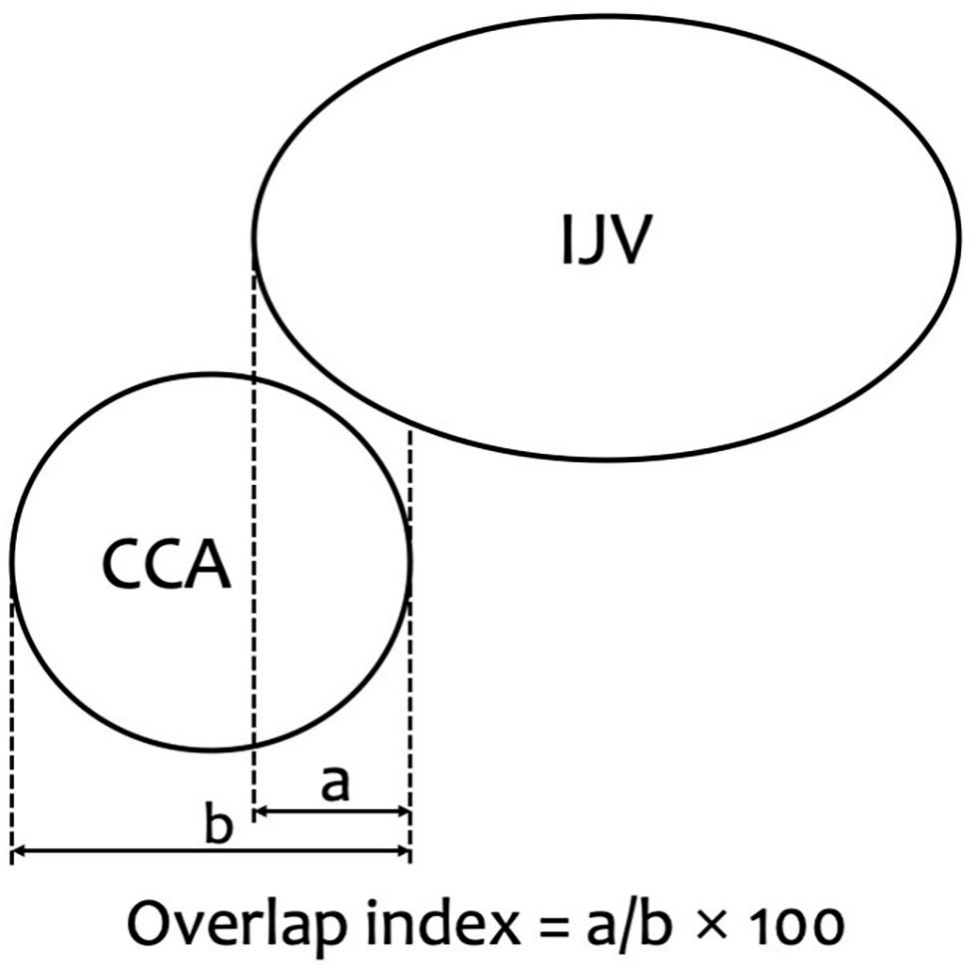
Overlap index is indicated. Overlap index = [overlap (mm)/CCA diameter (mm)] × 100. a, Overlap of common carotid artery (CCA) by the internal jugular vein (IJV) (mm). b, CCA diameter (mm).

The primary outcome of this study was overlap index of the IJV and the CCA before and after airway device placement. The overlap index was calculated as the ratio of the overlapping length of the IJV to the horizontal diameter of the CCA. The calculation formula is as follows: Overlap index = [IJV overlap length (mm)/CCA diameter (mm)] × 100 (Figure 1). As the index increases, the degree of overlap also increases. The CCA diameter and overlap length of the CCA and the IJV were measured using the in-built software of the machine.

**Figure 2:**
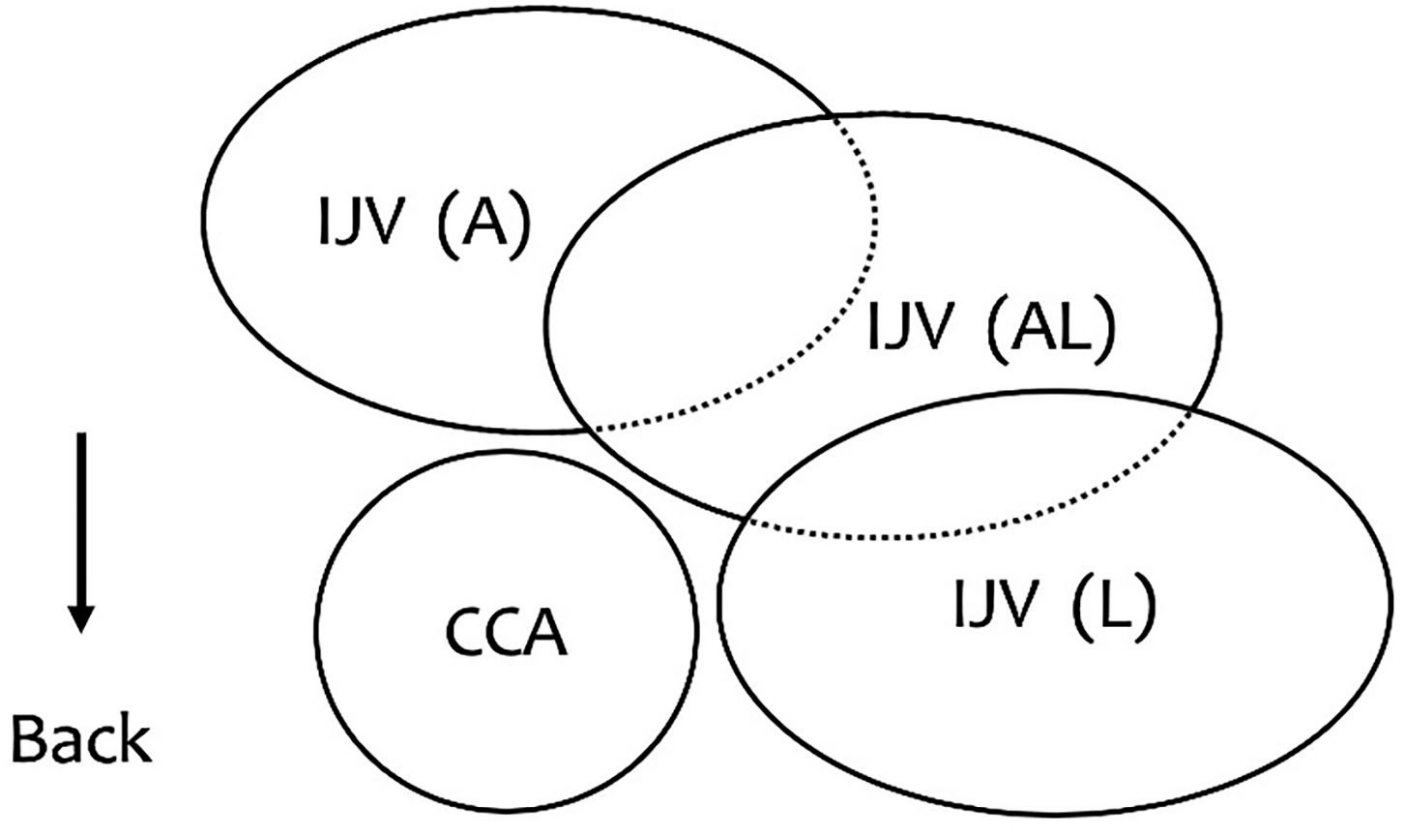
The relationship between the IJV and the CCA. A (anterior): The IJV was completely overlapping the CCA. AL (anterolateral): The IJV was partially covering the CCA. L (lateral): The IJV was alongside the CCA.

The secondary outcome was the IJV anatomical position in relationship to the CCA under ultrasonography; it was noted as anterior (A), anterolateral (AL), and lateral (L) (Figure 2). The classification is based on the overlap index of the IJV and the CCA; if the overlap index is 100, 1 to 99, 100, the anatomic position relationship is classified as A, AL, and L, respectively. The changes in anatomical position between the IJV and the CCA under ultrasonography also need to be observed.

## Statistics

Based on a pilot study we conducted, to detect a 20% difference in overlap index of the IJV and the CCA before and after airway device placement within each group, and ensure that the sample sizes of the study groups would support a valid comparison, a power analysis was performed (α = 0.05, β = 0.20); 40 patients were needed in each group. Metric variables were expressed as mean ± SD, and categorical variables were presented as number of patients and frequency (percent). The *t*-test was used for metric variables, and Fisher's exact test was used to analyze categorical variables since minimum expected count <5.

All data were analyzed using the SPSS for Windows software package (ver. 21.0; SPSS, Inc., Chicago, IL, USA). A *P*-value <0.05 was considered to indicate statistical significance.

## Results

In total, 101 patients were recruited to this study. Nine patients were excluded because of poor image quality. The investigators analyzed the images obtained from 44 patients in Group ETT and 48 patients in Group LMA. There were no adverse events or complications occurred in any of the patients.

The demographics of the patients are shown in Table 1. There was no significant difference between the demographic characteristics of patients in Group ETT and Group LMA.

**Table 1:** Demographic characteristics of the patients.

	**Group ETT (*n* = 44)**	**Group LMA (*n* = 48)**	***P*-value**
Age (month)	26.16 ± 18.92	26.98 ± 14.62	0.818
Height (cm)	86.61 ± 18.54	89.72 ± 12.13	0.350
Weight (kg)	12.15 ± 4.43	13.46 ± 4.36	0.157
Gender			1
Male	37	40	
Female	7	8	

*Metric values are expressed as mean ± SD. Data of gender are expressed as n*.

Before airway device insertion, as the head rotated 30° to the contralateral side, the overlap index increased significantly on the right side of the neck compared to the neutral head position (*P* = 0.023). However, there was no significant difference in the overlap index on the left side of the neck (*P* = 0.217).

Overlap indexes of the IJV and the CCA in the neutral head position and 30° head rotated position before and after airway device insertion are shown in Table 2. In Group ETT, there was no significant difference in the overlap indexes after intubation in the neutral head position or 30° head rotated position on either side. In Group LMA, the overlap indexes were increased significantly after LMA insertion in the neutral head position on both sides. Likewise, the overlap indexes were increased significantly after LMA insertion in the 30° head rotated position on both sides. Compared with the head in the neutral position before intubation, the overlap index was significantly increased when the head rotated 30° to the opposite side after LMA insertion.

**Table 2:** Overlap indexes before and after airway device insertion.

	**Neutral head position**		**30° head rotated position**		***P*****-value**			
	**Before insertion**	**After insertion**	**Before insertion**	**After insertion**	***P*^**a**^**	***P*^**b**^**	***P*^**c**^**	***P*^**d**^**
Group ETT (*n* = 44)								
Right side	21.18 ± 16.76	22.95 ± 14.64	24.73 ± 16.32	26.50 ± 16.80	0.599	0.618	0.294	0.141
Left side	31.58 ± 23.45	27.62 ± 20.38	34.23 ± 24.91	29.50 ± 24.12	0.400	0.368	0.695	0.682
Group LMA (*n* = 48)								
Right side	18.48 ± 13.21	39.70 ± 27.00	25.03 ± 14.19	57.76 ± 35.29	0.000	0.000	0.006	0.000
Left side	27.93 ± 18.46	45.98 ± 32.10	33.21 ± 21.42	64.68 ± 35.60	0.001	0.000	0.008	0.000

Values are expressed as mean ± SD.

a After airway device insertion vs. before airway device insertion in neutral head position.

b After airway device insertion vs. before airway device insertion in 30° head rotated position.

c After airway device insertion in 30° head rotated position vs. in neutral head position.

d *After airway device insertion in 30° head rotated position vs. before airway device insertion in neutral head position*.

The IJV anatomical position in relationship to the CCA in the neutral head position and 30° head rotated position under ultrasonography before and after airway device insertion is shown in Table 3. In Group ETT, before and after intubation, the most common positional relationship between the IJV and the CCA was AL in both the right side and left side in the neutral head position. Likewise, the AL position was still the most common position relationship between the IJV and the CCA before and after intubation in the 30° head rotated position. In Group LMA, in the neutral head position, the most common positional relationship between the IJV and the CCA was AL in both the right side and left side before and after LMA insertion, but the A position increased significantly after LMA insertion in the left side. In the 30° head rotated position, there was a significant increase to the A position after LMA insertion in both the right side and left side.

**Table 3:** Position of IJV relative to CCA before and after airway device insertion.

	**Relationship of IJV and CCA**	**Neutral head position**			**30° head rotated position**		
		**Before insertion**	**After insertion**	***P*-value**	**Before insertion**	**After insertion**	***P*-value**
Group ETT (*n* = 44)							
Right side	Anterior	0 (0)	0 (0)	1	0 (0)	0 (0)	1
	Anterolateral	39 (88.64)	40 (99.91)		41 (93.18)	41 (93.18)	
	Lateral	5 (11.36)	4 (9.09)		3 (6.82)	3 (6.82)	
Left side	Anterior	2 (4.55)	1 (2.27)	0.782	2 (4.55)	2 (4.55)	1
	Anterolateral	38 (86.36)	40 (99.91)		39 (88.64)	39 (88.64)	
	Lateral	4 (9.09)	3 (6.82)		3 (6.82)	3 (6.82)	
Group LMA (*n* = 48)							
Right side	Anterior	0 (0)	5 (10.42)	0.109	0 (0)	17 (35.42)	0.000
	Anterolateral	45 (93.75)	41 (85.42)		47 (97.92)	28 (58.33)	
	Lateral	3 (6.25)	2 (4.17)		1 (2.08)	3 (6.25)	
Left side	Anterior	0 (0)	9 (18.75)	0.004	1 (2.08)	23 (47.92)	0.000
	Anterolateral	43 (89.58)	36 (75.00)		42 (87.50)	24 (50.00)	
	Lateral	5 (10.42)	3 (6.25)		5 (10.42)	1 (2.08)	

*Values are expressed as no. (%)*.

**Figure 3:**
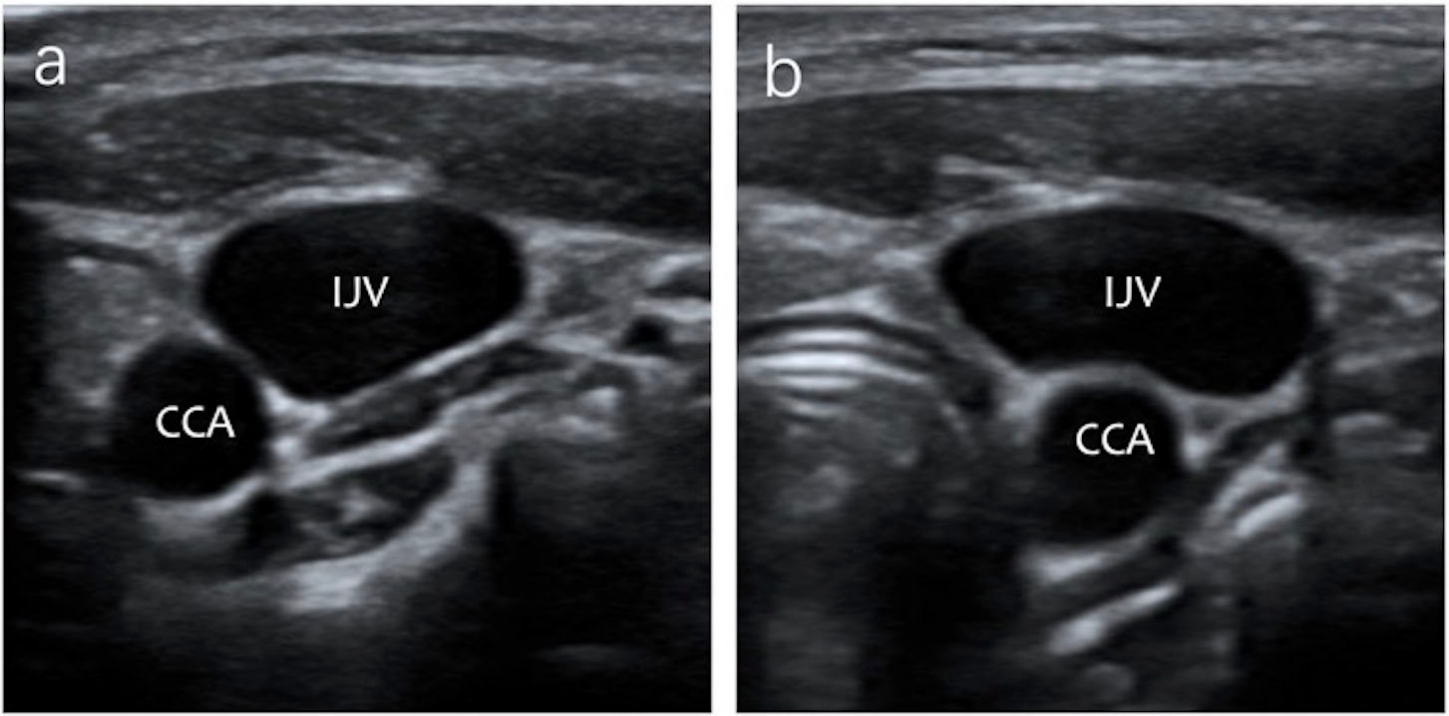
The IJV anatomical position in relationship changed from AL to A in a patient under ultrasound images after LMA insertion. **(a)** The anatomic position relationship is AL under ultrasonography before LMA insertion. **(b)** The anatomic position relationship is A under ultrasonography after LMA insertion.

The changes in anatomical position between the IJV and the CCA under ultrasonography after airway device insertion are shown in Table 4. In Group ETT, the main positional relationship between the IJV and the CCA did not change after intubation, either in the neutral head position or in the 30° head rotated position. In Group LMA, the change from AL to A was increased after LMA insertion, especially in the 30° head rotated position. Figure 3 shows that the anatomic position relationship changed from AL to A in a patient under ultrasonography after LMA insertion.

**Table 4:** Changes in anatomical position between the IJV and the CCA after airway device insertion.

	**Group ETT (*****n*** **= 44)**				**Group LMA (*****n*** **= 48)**			
	**Neutral head position**		**30° head rotated position**		**Neutral head position**		**30° head rotated position**	
	**Right side**	**Left side**	**Right side**	**Left side**	**Right side**	**Left side**	**Right side**	**Left side**
A → A	0 (0)	0 (0)	0 (0)	1 (2.27)	0 (0)	0 (0)	0 (0)	1 (2.08)
A → AL	0 (0)	2 (4.55)	0 (0)	1 (2.27)	0 (0)	0 (0)	0 (0)	0 (0)
A → L	0 (0)	0 (0)	0 (0)	0 (0)	0 (0)	0 (0)	0 (0)	0 (0)
AL → A	0 (0)	1 (2.27)	0 (0)	1 (2.27)	5 (10.42)	9 (18.75)	17 (35.42)	22 (45.83)
AL → AL	38 (86.36)	36 (81.82)	39 (88.64)	36 (81.82)	39 (81.25)	33 (68.75)	27 (56.25)	20 (41.67)
AL → L	1 (2.27)	1 (2.27)	2 (4.55)	2 (4.55)	1 (2.08)	1 (2.08)	3 (6.25)	0 (0)
L → A	1 (2.27)	0 (0)	0 (0)	0 (0)	0 (0)	0 (0)	0 (0)	0 (0)
L → AL	2 (4.55)	2 (4.55)	2 (4.55)	2 (4.55)	2 (4.17)	3 (6.25)	1 (2.27)	4 (8.33)
L → L	2 (4.55)	2 (4.55)	1 (2.27)	1 (2.27)	1 (2.08)	2 (4.17)	0 (0)	1 (2.27)

*Values are expressed as no. (%)*.

The change in anatomical position between the IJV and the CCA under ultrasonography after 30° head rotation is shown in Table 5. In Group ETT, the main positional relationship between the IJV and the CCA did not change after 30° head rotation, either before or after airway device insertion. In group LMA, the change from AL to A was increased after LMA insertion and 30° head rotation.

**Table 5:** Changes in anatomical position between the IJV and the CCA after 30° head rotation.

	**Group ETT (*****n*** **= 44)**				**Group LMA (*****n*** **= 48)**			
	**Before insertion**		**After insertion**		**Before insertion**		**After insertion**	
	**Right side**	**Left side**	**Right side**	**Left side**	**Right side**	**Left side**	**Right side**	**Left side**
A → A	0 (0)	0 (0)	0 (0)	1 (2.27)	0 (0)	0 (0)	2 (04.17)	9 (18.75)
A → AL	0 (0)	2 (4.55)	0 (0)	0 (0)	0 (0)	0 (0)	0 (0)	0 (0)
A → L	0 (0)	0 (0)	0 (0)	0 (0)	0 (0)	0 (0)	0 (0)	0 (0)
AL → A	0 (0)	2 (4.55)	0 (0)	1 (2.27)	0 (0)	1 (2.08)	13 (27.08)	14 (29.17)
AL → AL	37 (84.09)	35 (79.55)	37 (84.09)	28 (58.33)	44 (91.67)	40 (83.33)	27 (56.25)	21 (43.75)
AL → L	2 (4.55)	1 (2.27)	3 (6.82)	1 (2.77)	1 (2.08)	2 (4.17)	1 (2.27)	1 (2.27)
L → A	0 (0)	0 (0)	0 (0)	0 (0)	0 (0)	0 (0)	0 (0)	0 (0)
L → AL	4 (9.09)	2 (4.55)	4 (9.09)	1 (2.27)	3 (6.25)	2 (4.17)	1 (2.27)	3 (6.25)
L → L	1 (2.27)	2 (4.55)	0 (0)	2 (4.55)	0 (0)	3 (6.25)	1 (2.27)	0 (0)

*Values are expressed as no. (%)*.

## Discussion

During IJV catheterization, the CCA injury is a serious complication (11). Due to small vascular diameters and the proximity of the IJV and the CCA, the incidence of failure and the CCA injury during IJV catheterization in infants and children are high (12). After insertion of LMA, the anatomical position relationship between the IJV and the CCA may change, which increases both the difficulty of IJV catheterization and the risk of accidentally injuring the CCA (9). In this prospective observational study, we evaluated the impact of airway device (ETT or LMA) and 30° head rotation on the overlap index of the IJV and the CCA using ultrasonography, and its effect on the anatomical relationship between the IJV and the CCA.

In this study, we adopted some measures to increase the CSA of the IJV. Verghese et al. (13) reported that the IJV enlarged in the 15° Trendelenburg position. Sargin et al. (14) showed that using a 10-mL/kg tidal volume in pediatric patients could make the right IJV achieve the greatest size and caused no difference in the CCA overlap. An et al. (15) demonstrated that the application of positive end-expiratory pressure at 10 and 15 cm H2O increases the size of the right IJV in patients receiving mechanical ventilation with LMA insertion, but the overlap index of the IJV and the CCA increased as well, so this strategy was not adopted. There are conflicting results regarding the optimal angle of the head rotated to the contralateral side after airway devices insertion in children and adults (1, 7, 9, 10, 12). We adopted the 30° head rotated position because the overlap percentage of the IJV and the CCA likely increases at rotation angles in excess of 30°. Children have smaller necks than adults, so we only measured one point, the level of the cricoid cartilage.

The incidence of both anterior and posterior wall puncture of the IJV in pediatric patients during catheterization is obviously higher than in adults (16). When the IJV overlapped the CCA, both anterior and posterior wall puncture may increase the opportunity of accidental arterial puncture (1). The effects of head rotation and airway device type on the overlap index of the IJV and the CCA are still inconclusive.

Wang et al. (17) compared the overlap percentage of the CCA by the IJV in 156 adult patients under 0, 45, and 90° head rotation. They found that as the head was turned, the percent overlap increased. Sulek et al. (18) studied the percent overlap of the CCA and the IJV at 0, 40, and 80° head rotation in 12 volunteers aged 18–60 years. They demonstrated that the percent overlap of the CCA and the IJV increased significantly at 40 and 80° head rotation to both sides of the neck. Vosylius et al. (19) studied the overlapping angle of the IJV by the CCA in 82 patients under neutral head position and at a head rotation of 15, 30, 45, and 60° to the left. They found that the overlapping angle increased significantly at 45 and 60° head rotation compared with the neutral head position. Gwak et al. (1) reported that 40° contralateral head rotation increased the right IJV size with less resulting of the CCA overlap than at 80° in infants and young children. So they suggest that 40° head rotation is optimal for right IJV catheterization in pediatric patients. Our study did not turn the head of patients in excess the angle of 30°. Before airway device insertion, the overlap index at the 30° head rotation position was significantly increased compared to the neutral head position on the right side of the neck but not on the left side. The overlap index of the left side did not increase significantly. It was probably related to the anatomical difference between the left and right sides, but further studies are still needed to explore the specific reasons.

Ozcelik et al. (10) studied the overlap percentage of the CCA by the IJV after airway device insertion in a neutral head position and 40° head rotation position in 92 subjects aged 0–17 years. In contrast to our study, they demonstrated that LMA with the 40° head rotation increased the overlap percentage of the CCA by the IJV and ETT decreased the overlap percentage. There was a difference in the degree of head rotation, and they turned the head of the subject 40° to the opposite side, whereas we turned 30°. The ages of their study patients ranged from 0 to 17 years, while ours ranged from 1 month to 6 years.

Matsuda and Arai (9) studied the degree of overlap of the right IJV and the CCA before and after LMA insertion in infants and children with their heads rotated 30° to the left; they showed that there was no remarkable change in the overlap index after LMA insertion. However, they did not compare patients with their heads in the neutral and rotated position before and after LMA insertion. Our results showed that there were no significant changes in the overlap index of the IJV and the CCA after ETT intubation and 30° head rotation in infants and children. However, insertion of LMA caused a significant increase in the overlap index of the IJV and the CCA in patients with neutral and 30° head rotated position on both sides of the neck. These findings indicate that LMA insertion is an important factor in the increase of overlap index.

Kim et al. (20) studied the overlap index of the CCA and the IJV after LMA insertion with different angles of head rotation in adult patients. They demonstrated that the overlap index increased significantly with greater head rotation degrees after LMA insertion. Our study showed that after LMA insertion, the head rotated 30° to the contralateral side significantly increased the overlap index of the IJV and the CCA on both sides compared to the head in the neutral position. This finding is consistent with the previous study. This suggests that increase in overlap index should be noted when the heads of patients were rotated to the contralateral side after LMA insertion.

The present study also evaluated the impact of airway device (ETT or LMA) and 30° head rotation on IJV location change. The results showed that the most common anatomical position of the IJV relative to the CCA was AL on both sides of the neck. Hong et al. (12) studied 200 pediatric patients and demonstrated that the relative position of the IJV to the CCA is AL on both sides of the neck (right, 58.5%; left, 67.5%). Moreover, Ozcelik et al. (10) showed that the AL is the most frequently anatomical position both right and left sides of the neck and did not change after LMA insertion. Our finding is consistent with these previous studies.

In our study, after ETT insertion and 30° head rotation, the position of the IJV did not change significantly. The A position was increased after LMA insertion on the left side and significantly increased after 30° head rotation on both sides of the neck. Similarly, the change from AL position to A position was increased after LMA insertion, especially in the 30° head rotated position. In a previous study, Takeyama et al. (7) reported a similar result in adults; one possible reason is that when the head rotated after LMA insertion, the CCA tends to be subject to external pressure from the inflated cuff, causing it to lean toward the dorsal side of the IJV. Furthermore, the LMA insertion may displace the sternocleidomastoid muscle, which makes palpating the CCA difficult (20). So, the usual attempts to catheterize the IJV just lateral to the CCA may result in failure. This suggests that the operator should be aware of the increased risk of arterial punctures after LMA insertion and 30° head rotation.

Measures should be taken to improve the success rate of the IJV catheterization and reduce the risk of CCA injury. Despite the greater technical difficulty of IJV catheterization because of the needle insertion angle which required to be more acute while in the neutral head position, Hong et al. (12) recommend minimizing the degree of head rotation when catheterizing the IJV, especially in infants and children. Arai et al. (21) demonstrated the overlap index between the IJV and the CCA which increased when the head rotated in infants and children, so they did not recommend head rotation during IJV catheterization. Riley et al. (22) recommended deflating the cuff of the LMA to improve the success rate of IJV catheterization, but we believe this could lead to air leakage, making ventilation inadequate. Based on the results of the present study, we recommend minimizing the degree of head rotation during IJV catheterization in infants and children after LMA placement. However, the needle and catheter would contact the mandible during the catheterization, which makes the procedure difficult, so a small angle rotation of the head is acceptable. In addition, it is necessary to confirm using an ultrasound scanner during IJV catheterization.

Our study has several limitations. First, the subjects of the present study were infants and children, but we did not divide them into infant and child groups. Second, because the ultrasound probe inevitably compressed the vein, some measurement errors may occur. Third, double blindness is not possible because the ultrasound examiner was aware of the head position and the inserted airway device, which may have affected the results. Fourth, there are many ways to increase the CSA of the IJV, such as Valsalva maneuver, passive leg elevation, and liver compression (13); if we adopted these strategies, the results may have been different. Further studies could examine the validity of this study in clinical practice and divide the subjects into infant and child groups.

## Conclusions

The overlap index increased significantly in both sides of the neck after LMA placement in the neutral head position, especially in the 30° head rotated position. The IJVs after LMA placement had a tendency to become anterior to the CCA when the head of the patient rotated in the opposite direction. We recommend minimizing the degree of head rotation and using an ultrasound scanner during IJV catheterization in infants and children after LMA placement.

## Data Availability Statement

The raw data supporting the conclusions of this article will be made available by the authors, without undue reservation.

## Ethics Statement

The studies involving human participants were reviewed and approved by First Hospital of Jilin University. Written informed consent to participate in this study was provided by the participants' legal guardian/next of kin.

## Author Contributions

YD helped to design the study, operate the ultrasound machine, collect ultrasonic images, and write the manuscript. JW helped to analyze the data and write the manuscript. LJ helped to measure the data of ultrasonic images. CL and HM helped to design the study and write the manuscript. SD helped to design the study, conducted the study, and wrote the manuscript. All authors contributed to the article and approved the submitted version.

## Conflict of Interest

The authors declare that the research was conducted in the absence of any commercial or financial relationships that could be construed as a potential conflict of interest.
